# Pulmonary sclerosing pneumocytoma mimicking a low-grade primary malignancy: A case report

**DOI:** 10.1016/j.ijscr.2024.109668

**Published:** 2024-05-01

**Authors:** Tomohide Ando, Tomonari Oki, Shuhei Iizuka, Yoshiro Otsuki, Toru Nakamura

**Affiliations:** a Department of General Thoracic Surgery, Seirei Hamamatsu General Hospital, 2-12-12, Sumiyoshi, Naka-ku, Hamamatsu-city, Shizuoka 430-8558, Japan; b Department of Pathology, Seirei Hamamatsu General Hospital, 2-12-12, Sumiyoshi, Naka-ku, Hamamatsu-city, Shizuoka 430-8558, Japan

**Keywords:** Pulmonary sclerosing pneumocytoma, Volume doubling time, Lung neoplasms

## Abstract

**Introduction:**

Pulmonary sclerosing pneumocytoma (PSP) is a rare benign tumor classified as a pulmonary adenoma. It presents as a solitary pulmonary nodule without any specific findings, often posing a diagnostic challenge. We herein present a case of a PSP with a short volume doubling time (VDT) comparable to low-grade pulmonary malignancies.

**Case presentation:**

A 27-year-old female presented to the emergency department with a fever that had persisted for the past two days. An incidental finding on chest screening computed tomography (CT) revealed a 9 mm pulmonary nodule with a round shape and smooth margin, suggestive of a benign etiology.

Follow-up CT one year later revealed an enlarged nodule exhibiting a VDT of 249 days. A thoracoscopic lingulectomy was performed, and the histopathological examination revealed papillary and diffuse proliferation of epithelial-like cells. The epithelial cells were positive for cytokeratin (CKAE1/AE3) and thyroid transcription factor 1 (TTF1), whereas the stromal cells were positive for TTF1 but negative for CKAE1/AE3. Those results were consistent with the diagnosis of a PSP.

**Discussion:**

PSPs typically present as incidental pulmonary nodules with no specific findings, often posing a diagnostic challenge. The radiographic features of PSPs have mainly been explored based on the morphological findings and metabolic activity, with limited research on their growth rate, represented by the VDT.

**Conclusion:**

PSPs may exhibit rapid growth, demonstrating a short VDT similar to that of low-grade pulmonary malignancies. Comprehensive diagnostic testing not based solely on the growth rate for this rare condition is essential.

## Highlights

• Pulmonary sclerosing pneumocytomas (PSPs) is difficult to diagnose due to the lack of specific radiological findings.
• Radiographic features of PSPs have mainly been limited research on their growth rate.
• Solitary pulmonary nodules with a rapid growth pattern typically indicate a primary malignancy.
• PSPs may exhibit a short volume doubling time similar to that of low-grade pulmonary malignancies.

## 1 Introduction

Pulmonary sclerosing pneumocytoma (PSP) is a rare benign tumor of the lung previously known as sclerosing hemangioma and classified as a pulmonary adenoma [1,2]. It presents as a solitary pulmonary nodule without any specific findings, often posing a diagnostic challenge. While its radiological characteristics have been discussed in the literature, there have been limited reports addressing the volume doubling time (VDT) thus far. In this report, we present a case of a PSP with a VDT comparable to malignancies such as carcinoid tumors. This work has been reported in line with the SCARE criteria [3].

## 2 Case presentation

**Fig. 1:**
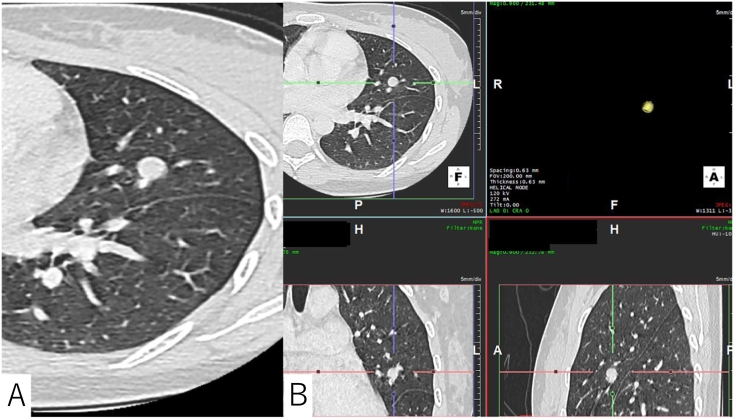
A: A chest CT showing a 9 mm pulmonary nodule with a round shape and smooth margin in the left upper lobe. B: The volume of the nodule was estimated to be 425 mm3 based on the three-dimensional CT volume rendering reconstruction.

**Fig. 2:**
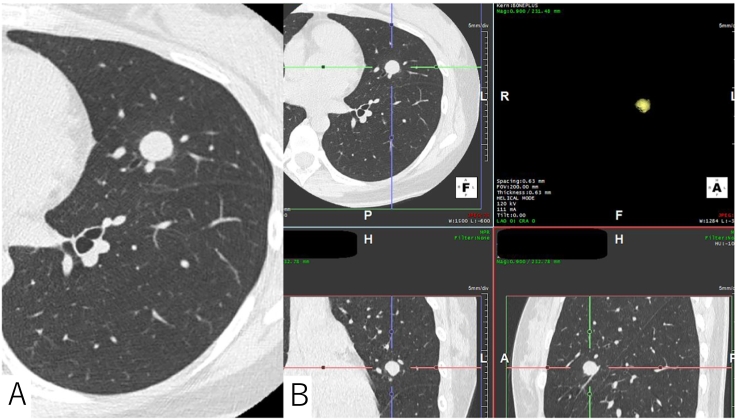
A: The nodule enlarged to a maximum size of 14 mm one year later. B: The nodule enlarged to a volume of 1050 mm^3^.

A 27-year-old woman, who had never smoked, presented with a fever persisting for the past two days. The patient had no relevant past medical history or family history. A physical examination revealed left costovertebral angle tenderness and a urinalysis revealed the presence of pyuria. There were no other indications suggestive of an obvious fever source. The diagnosis was pyelonephritis, and intravenous ceftriaxone was administered. An incidental finding on screening chest computed tomography (CT) revealed a 9 mm pulmonary nodule with a round shape and smooth margin in the left upper lobe (Fig. 1A). She reported no weight loss, hemoptysis, or shortness of breath. A three-dimensional CT scan obtained at a 0.63 mm thickness with volume rendering showed that the nodule volume was 425 mm^3^ (Fig. 1B). Volume rendering was obtained on Aquarius iNtuition 3D workstation (TeraRecon Inc., San Mateo, CA). Based on its radiological features, a benign pulmonary nodule was suspected and a radiographic follow-up was planned. One year later, the nodule enlarged to a maximum diameter of 14 mm (Fig. 2A) with an estimated volume of 1050 mm^3^ (Fig. 2B). No other pulmonary lesions or lymphadenopathies were observed. Due to the health insurance limitations, positron emission tomography was not accessible. A rapid growth pattern, with an estimated VDT of 249 days calculated using the Schwartz formula, prompted us to consider a malignancy [4]. Transbronchial biopsy was considered non-diagnostic because of the absence of a bronchial connection to the lesion, based on the thin-section CT. A surgical resection was planned for both diagnostic therapeutic purposes. The patient underwent a thoracoscopic lingulectomy successfully and was discharged uneventfully on the fifth postoperative day.

**Fig. 3:**
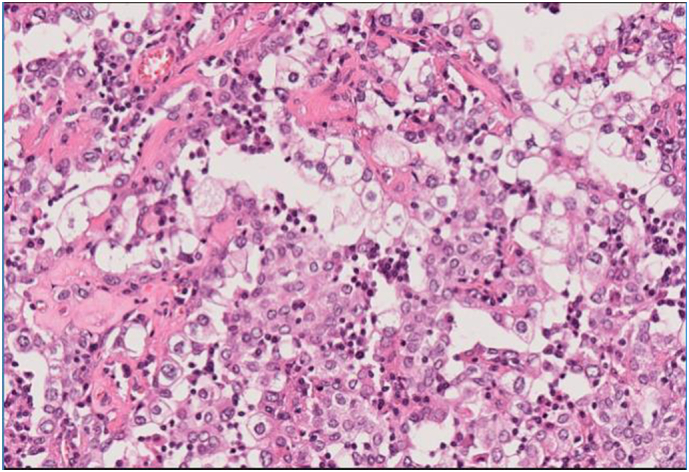
Histopathological examination showing papillary and diffuse proliferation of epithelial-like cells, accompanied by increased numbers of round, relatively large cells in the tissue stroma.

**Fig. 4:**
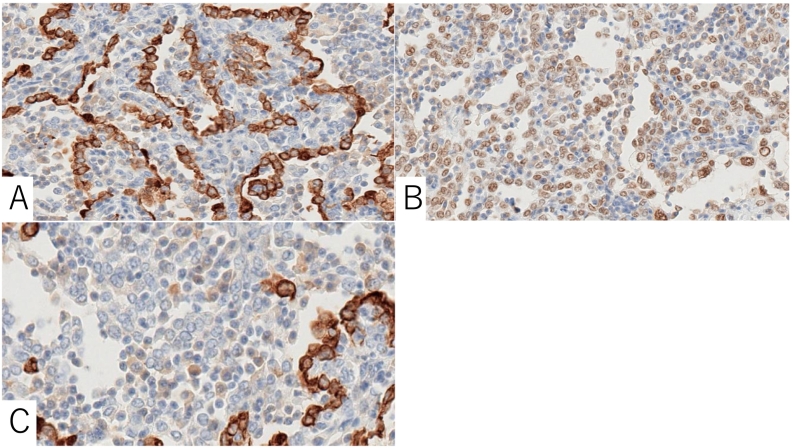
A: The epithelial cells were positive for CKAE1/AE3. B: The epithelial cells were positive for TTF1. C: The stromal cells were positive only for TTF1.

A histopathological examination revealed that the tumor was composed of a mixture of papillary, sclerotic, and solid patterns. The papillary area showed cores of fibrovascular sclerotic stroma and epithelioid cells lined by cuboidal epithelium. The solid area exhibited sheets of round to oval cells with pale eosinophilic cytoplasm (Fig. 3). Additionally, there was chronic infiltration of inflammatory cells, predominantly lymphocytes and plasma cells (Fig. 3). Immunohistochemical staining showed that the epithelial cells tested positive for cytokeratin (CKAE1/AE3) and thyroid transcription factor 1 (TTF1) (Fig. 4A and B), while the stromal cells were positive for TTF1 but negative for CKAE1/AE3 (Fig. 4C). No mitotic activity was not observed. Those results were consistent with a diagnosis of a PSP. She is currently disease free at 2 months since surgery.

## 3 Discussion

PSPs typically present as incidental pulmonary nodules with no specific findings, and are often misdiagnosed as malignancies. The diagnostic dilemma with PSPs arises from their rarity and the lack of specific radiological findings, with correct diagnostic rates reported at a maximum of 30.3 % even in large case series [5]. A hamartoma was considered as a potential differential diagnosis due to its well-defined appearance in the present case, but it was excluded based solely on its rapid growth, which indicated potential malignancy. The possibility of a metastatic tumor was also ruled out due to its isolated occurrence, which is an atypical presentation for a metastatic lesion.

To date, the radiographic features of PSPs have mainly been explored based on the morphological findings and metabolic activity, with limited research on their proliferative capacity [5,6]. The CT findings in the present case revealed a solitary, round-shaped nodule with a smooth margin that remained unchanged after a one-year follow-up, consistent with that of a PSP. Because positron emission tomography was not available, we focused on the VDT to speculate on its growth rate.

A rapid growth rate indicated by a VDT of 249 days raised suspicion of a malignancy. Except for the rapid enlargement, the radiological findings remained typical of a PSP. The epidemiological profile, especially among middle-aged Asian women, further substantiated the diagnosis [[7], [8], [9]].

The volume doubling time refers to the time required for a tumor to double in volume and is used to differentiate it between benign and malignancy [10]. While a long median doubling time of 965 days indicates a slow-growing nature and indolent behavior of a PSP, a short VDT of 374 days may also represent its biological diversity in the literature [[11], [12], [13], [14]]. The shortest VDT of 249 days in the present case seemed comparable to that of a low-grade malignancy such as a carcinoid tumor [[15], [16], [17]]. As a result, we misdiagnosed this case as a malignancy based solely on the short VDT. A histopathological examination in the present case revealed a typical finding compatible with a PSP, showing no mitotic activity. The pathogenesis of such a rapid growth remains unclear. Some PSPs may manifest with malignant characteristics, including metastases and even a potential fatality, despite their benign nature, demonstrating a broad spectrum of their biological features [18,19]. The report suggesting that regional lymph node metastases do not impact prognosis contributes not only to diagnostic but also to therapeutic challenges [20]. Besides its rarity and the lack of specific radiological findings, this biological diversity may lead to diagnostic confusion of a PSP.

## 4 Conclusion

PSPs may exhibit rapid growth, demonstrating a short VDT similar to that of low-grade pulmonary malignancies. The present case illustrated the diagnostic challenge of PSPs based solely on the growth rate and also underscored the importance of a comprehensive diagnostic workup for this rare entity.

## Consent for publication

Written informed consent was obtained from the patient for publication of this case report and accompanying images. A copy of the written consent is available for review by the Editor-in-Chief of this journal on request.

## Ethical approval

Ethical Approval was not required because the manuscript was not a research study and we only have the patient consent for writing and other forms of publication.

## Funding

Not applicable.

## Author contribution

AT wrote this paper. YO reviewed the pathological findings. All authors read and approved the final manuscript.

## Guarantor

Toru Nakamura.

## Research registration number

Not applicable.

## Provenance and peer review

Not commissioned, externally peer-reviewed.

## Conflict of interest statement

The authors declare that they have no competing interests.

## Data Availability

Not applicable.
